# Efficacy of natural killer T and gammadelta T cells in mesothelin-targeted immunotherapy of pancreatic cancer

**DOI:** 10.3389/fimmu.2025.1524899

**Published:** 2025-02-10

**Authors:** Yuankui Zhu, Yaxi Yang, Linghe Yue, Lei Wan, Xuqian Ma, Qing Yang, Xuan Tian, Yuguan Li, Ke Wang, Shaozhong Wei, Dianbao Zuo, Mingqian Feng

**Affiliations:** ^1^ College of Biomedicine and Health, Huazhong Agricultural University, Wuhan, Hubei, China; ^2^ Department of Gastrointestinal Oncology Surgery, Hubei Cancer Hospital, Tongji Medical College, Huazhong University of Science and Technology, Wuhan, Hubei, China; ^3^ Department of Gastrointestinal Oncology Surgery, Colorectal Cancer Clinical Research Center of Hubei Province, Wuhan, Hubei, China; ^4^ Department of Gastrointestinal Oncology Surgery, Colorectal Cancer Clinical Research Center of Wuhan, Wuhan, Hubei, China; ^5^ Research Center for Translational Medicine, Hubei Key Laboratory of Wudang Local Chinese Medicine Research, Hubei Provincial Clinical Research Center for Parkinson’s Disease at Xiangyang No.1 People’s Hospital, Hubei University of Medicine, Xiangyang, China; ^6^ College of Life Science & Technology, Huazhong Agricultural University, Wuhan, Hubei, China

**Keywords:** NKT, γδT, PBMCs, bispecific antibody, pancreatic tumor

## Abstract

Current pancreatic cancer immunotherapy focused on alphabeta (αβ) T cells, either through CD3-engaged bispecific antibodies or CAR-T. Despite their promise, dose-limited toxicity (DLT) remains a challenge in clinical practice. In light of these concerns, there is a growing interest in exploring alternative T cell types, natural killer T (NKT) cells and gammadelta (γδ) T cells, that possess the capacity to lyse tumors while potentially offering a safer therapeutic profile with fewer side effects. These cells present a compelling alternative that warrants a comprehensive evaluation of their therapeutic potential and safety profile. This study employed a MSLN/CD3 bispecific antibody to compare the anti-tumor activity of NKT and γδT cells with peripheral blood mononuclear cells (PBMCs) as controls, both *in vitro* and *in vivo*. This study demonstrated that MSLN/CD3 BsAb effectively activated and recruited PBMCs, NKT and γδT. Furthermore, under the influence of MSLN/CD3 BsAb, γδT and NKT cells exhibited notably superior anti-tumor activity compared to PBMCs, both *in vitro* and *in vivo*, while demonstrating low cytokine release. γδT cells showed almost negligible toxic side effects. In addition, the systemic administration of NKT and γδT cells activators, α-galactosylceramide (α-GalCer) and Zoledronate, could enhance the anti-tumor effect of MSLN/CD3 bsAb, with no apparent toxicity. NKT and γδT cells are promising synergistic therapeutic cell types that may overcome the limitations of CD3 bispecific antibodies in pancreatic tumor treatments, offering a new perspective for clinical applications in immunotherapy.

## Introduction

Pancreatic cancer is one of the deadliest malignancies, with a five-year survival rate of less than 10%. In 2022, over 510,000 new cases were reported globally, with approximately 467,000 deaths, making it the 12th most common cancer (1). The high mortality rate is largely due to late-stage diagnosis and limited effective treatment options (2). Current treatments for pancreatic cancer include surgery, chemotherapy, and radiation therapy. However, these approaches offer only marginal improvements in survival, especially for patients with metastatic pancreatic cancer (3). Immunotherapy has emerged as a promising strategy, yet its application in pancreatic cancer faces significant challenges due to the immunosuppressive tumor microenvironment (TME).

Currently, monoclonal antibodies (mAb), cancer vaccines, immune checkpoint blockade, and CAR-T and bispecific antibodies (BsAb) are powerful tools for cancer immunotherapy (4, 5). BsAbs, such as blinatumomab targeting CD19 and CD3, have demonstrated efficacy in hematological malignancies (6). Combining MSLN-targeting antibodies with CD3 bispecific antibodies has emerged as a promising therapeutic approach in immuno-oncology. The rationale is to redirect T-cells to tumor cells overexpressing MSLN, leading to T-cell-mediated cytotoxicity and tumor cell elimination. Preclinical studies have demonstrated the efficacy of MSLN/CD3 bispecific antibodies in targeting MSLN-expressing tumors. These studies showed significant tumor regression in xenograft models, highlighting the potential of this strategy in various malignancies (7, 8). However, their effectiveness in solid tumors, including pancreatic cancer, is limited by factors such as the TME, intrinsic tumor resistance, and poor antibody penetration (9–12). Additionally, BsAbs can induce severe cytokine release syndrome (CRS), leading to life-threatening complications such as fever, hypotension, and multi-organ failure. Therefore, there is an urgent need to develop new strategies to enhance BsAb-mediated killing of solid tumors while minimizing their toxicity (13–15). Given these challenges, it is imperative to devise novel approaches to improve the efficacy of BsAbs in solid tumors and reduce associated toxicities.

Non-conventional T cells, such as γδT cells and NKT cells, have strong anti-infection, anti-tumor, immune tolerance and immune regulation functions (16–19). Based on their effective MHC-unrestricted cytotoxicity against different solid tumors, they have important implications for cancer immunotherapy (20, 21). Gamma delta T cells, especially the Vγ9Vδ2 subset, are the major subset of gamma delta T cells in human peripheral blood. They are characterized by non-MHC-restricted antigen recognition, abundant cytokine secretion capacity, and the ability to use various surface receptors and cytokines, such as NKG2D, TRAIL, FASL, TNF-α, IFN-γ, Granzyme B, and perforin, to initiate cytotoxicity against cancer cells. These indicate that they have high anti-tumor potential (22). These findings underscore their significant anti-tumor potential. To date, γδT cell transfer therapy has been explored in various cancers, including renal cell carcinoma, malignant leukemia, advanced lung cancer, among others. The majority of trials indicate favorable tolerability and safety profiles (23–25).

iNKT cells are an evolutionarily conserved innate T cell subset that express NK cell surface markers and invariant Vα24-Jα18 TCRα-chain (26). They are activated and characterized by their reactivity to self and microbial glycolipids presented by the monomorphic HLA-I-like molecule CD1d (27). A key advantage of NKT cells over conventional T cells may be their remarkable intrinsic anti-tumor activity, which is activated by glycolipids presented by CD1d on antigen-presenting cells through their endogenous T cell receptors (TCRs) (28, 29). α-GalCer is a widely studied CD1d ligand that induces IL-4 and interferon γ (IFN-γ) production in the TCR engagement of NKT cells. Despite the CD1d negativity of most solid tumors, the anti-tumor potential of NKT cells has been demonstrated in many cancer models (21, 30, 31).

In this study, bispecific antibodies were utilized to target different subsets of T cells (NKT, γδT cells), and the anti-tumor effects of PBMCs, NKT, and γδT cells were evaluated both *in vitro* and *in vivo*. *In vitro* experiments revealed that γδT cells demonstrated superior anti-tumor effects compared to PBMCs and NKT cells, along with lower levels of pro-inflammatory cytokine release. *In vivo* experiments further demonstrated the superior anti-tumor effects of γδT cells over NKT and PBMC cells, without inducing significant toxic side effects.

## Materials and methods

### Cell lines

The human embryonic kidney cell line 293F (RRID: CVCL_6642), along with three human mesothelin-expressing cell lines A431-H9 (a transfected A431 cell line stably expressing mesothelin), KLM-1 (RRID: CVCL_5146), and T3M4 (RRID: CVCL_4056) were utilized in this study. The mesothelin-negative A431 cell line (RRID: CVCL_0037) was also included. A431 and 293F were obtained from the China Center for Type Culture Collection, while KLM-1 and T3M4 were purchased from the American Type Culture Collection. All cells were cultured in DMEM medium (Invitrogen, Carlsbad, CA) supplemented with 10% FBS (HyClone, Logan, UT), 2mM L-glutamine and 1% penicillin-streptomycin (Invitrogen, Carlsbad, CA) and incubated in 5% CO2 with a balance of air at 37°C. All the cell lines were retrovirally transduced to express GFP/luciferase fusion protein.

### Antibody expression and purification

Mesothelin-targeted monoclonal antibody R47, developed in our laboratory from previous work (32), was utilized to construct the bispecific antibody. The bispecific antibody (MSLN/CD3 bsAb) format was R47scfv-hFc-G4S-OKT3scFv (shortened as scFv-hFc-scFv). The MSLN/CD3 bispecific antibody was expressed in HEK-293F cells in a secreted form. The bispecific antibody plasmids carrying the fusion gene were introduced into 293F cells using polyethyleneimine for transient expression. Six days post-transfection, the supernatant from the transfected cells was collected. Protein purification was performed using a Protein A column, followed by buffer exchange with a Sephadex G-25 desalting column. The purity of the preparation was assessed by SDS-PAGE.

### Isolation and culturing of lymphocyte populations

Human PBMCs were isolated from whole blood of healthy donors (Wuhan Blood Center) by Ficoll separation (Stem Cell Technologies, Vancouver, BC, Canada) according to the manufacturer’s instruction.

To establish NKT cell lines, PBMCs were cultured in RPMI 1640 containing 10% FBS the presence of α-GalCer (100 ng/ml) and recombinant interleukin-2 (rIL-2) (100 U/mL). α-GalCer is a potent ligand for NKT cells, and rIL-2 was used to promote the growth and activation of these cells. After 10–14 days, human NKT cells were restimulated by co-culture with irradiated PBMCs (typically irradiated at 25–30 Gy) that had been pulsed with α-GalCer (100 ng/mL) for 2–4 hours prior to the culture. This restimulation step was repeated every 2 weeks for a period of at least 1 month to maintain NKT cell expansion (33).

To establish γδT cell lines, PBMC were cultured in RPMI 1640 supplemented with 10% FBS (HyClone, Logan, UT), 1% L-glutamine, and 1% penicillin-streptomycin (Invitrogen, Carlsbad, CA). The cells were plated at a density of 1 × 10^6^ cells/mL in 6-well plates. γδT cells were activated by adding Zoledronate (5 μmol/L) (Adooq Bioscience), a bisphosphonate that induces the activation and expansion of γδT cells. Zoledronate was added to the culture medium at the beginning of the culture and refreshed twice a week. Every 2 days, the culture medium was supplemented with 100 U/mL rIL-2 support the proliferation of γδT cells. The culture period for the γδT cell lines lasted 14 to 21 days. After 14–21 days, γδT cells were harvested for further analysis or experimental procedures.

### Flow cytometry method

This research used a FACS Calibur (BD Biosciences, Franklin Lakes, NJ) to measure the fluorescence associated with live cells. All cells were harvested by detaching with trypsin-EDTA (ThermoFisher, Waltham, MA), washed by centrifugation, and resuspended in ice-cold PBS containing 5% BSA. The phenotype of NKT cells was assessed using Vα24-Jα18 mAb (6B11) followed by staining with a secondary goat anti-mouse-IgG-Cy5(Sangon biotch, China). The phenotype of γδT cells was assessed using TCR gamma/delta antibody (5A6.E9) followed by staining with a secondary goat anti-mouse-IgG-Cy5. Flow cytometry results were analyzed to determine the frequency of NKT and γδT cells in the sample. For the identification of CD3+ cells, PBMCs, NKT, and γδT cells were stained with the MSLN/CD3 bispecific antibody for 60 minutes at 4°C, followed by secondary staining with goat anti-mouse IgG-Cy5.

### T cell activation and cytokine analysis

Human PBMCs, NKT, or γδT cells were co-cultured with KLM-1 cells in a 10:1 effector-to-target cell ratio in the presence of MSLN/CD3 bispecific antibody (bsAb) for 24 hours in flat-bottom 96-well plates (BD Biosciences). To assess T cell activation, surface expression of CD69 was analyzed by flow cytometry, with CD69 serving as a marker of early T cell activation. Following the incubation, cytokines in the culture supernatants were collected and analyzed using human IL-1β and IL-6 ELISA kits (Mlbio, China), according to the manufacturer’s instructions, to quantify the secretion of pro-inflammatory cytokines.

### *In vitro* cytotoxicity assay

Cell growth and viability were assessed by firefly luciferases reporter assay. All the tested cancer cells were stably transfected to constitutively express a fire fly luciferase reporter gene (ffLuc2). One hundred microliters of stably transduced cells were seeded on a 96-well plate (5 × 10^3^ cells per well), followed by the addition of PBMC, NKT and γδT cells and bispecific antibodies at the indicated concentrations. After being incubated at 37°C for 72 h, cells were collected by spinning down, followed by lysis via two rounds of freeze-thaw. Released luciferase activity was measured to represent the cell viability.

### *In vivo* study

All animal experiments were conducted in accordance with the regulations of the Laboratory Animal Care Committee of Huazhong Agricultural University (HZAUMO-2023-0304). All the operations and experimental procedures were complied with the national standard of Laboratory Animal-Guideline for ethical review of animal welfare (GB/T 35892–2018). Female NSG mice, were purchased from Beijing Vital River Laboratory Animal Technology Co., Ltd and maintained under specific pathogen-free conditions. For *in vivo* therapeutic experiments, 1-2 million A431(H9) cells or five million KLM-1 were subcutaneously injected into NSG mice. Seven days after tumor engraftment, the tumor formed and reached the size of 100-200 mm^3^, approximately 5 × 10^6^ PBMCs, NKT or γδT and different concentrations of MSLN/CD3 bsAb were injected intravenously into tumor-bearing mice. PBMCs, NKT or γδT was administered twice a week and MSLN/CD3 bsAb was administered every three days. Tumor dimensions were measured every two days with a caliper and calculated by the following formula: Tumor volume (mm^3^) = (length × width × width) × 0.5.

### H&E staining

The sections obtained from organs (including brain, heart, liver, spleen, lung and kidney) from each group were paraffin-embedded and sliced into 4-µm per section. Then, the slides were baked at 65°C for 1 h, deparaffinized in xylene, rehydrated by graded ethanol, and stained with H&E successively.

### Statistical analysis

All statistical analyses were conducted using GraphPad Prism5 (GraphPad Software, Inc., La Jolla, CA). Comparison of two groups was performed using unpaired Student’s t-test (two tailed). Comparisons among three or more groups were performed using one- way analysis of variance. Two-way ANOVA analysis of variance was used for tumor growth curve. P<0.05 was considered statistically significant.

## Results

### Production and functional testing of NKT and γδT cells

**Figure 1:**
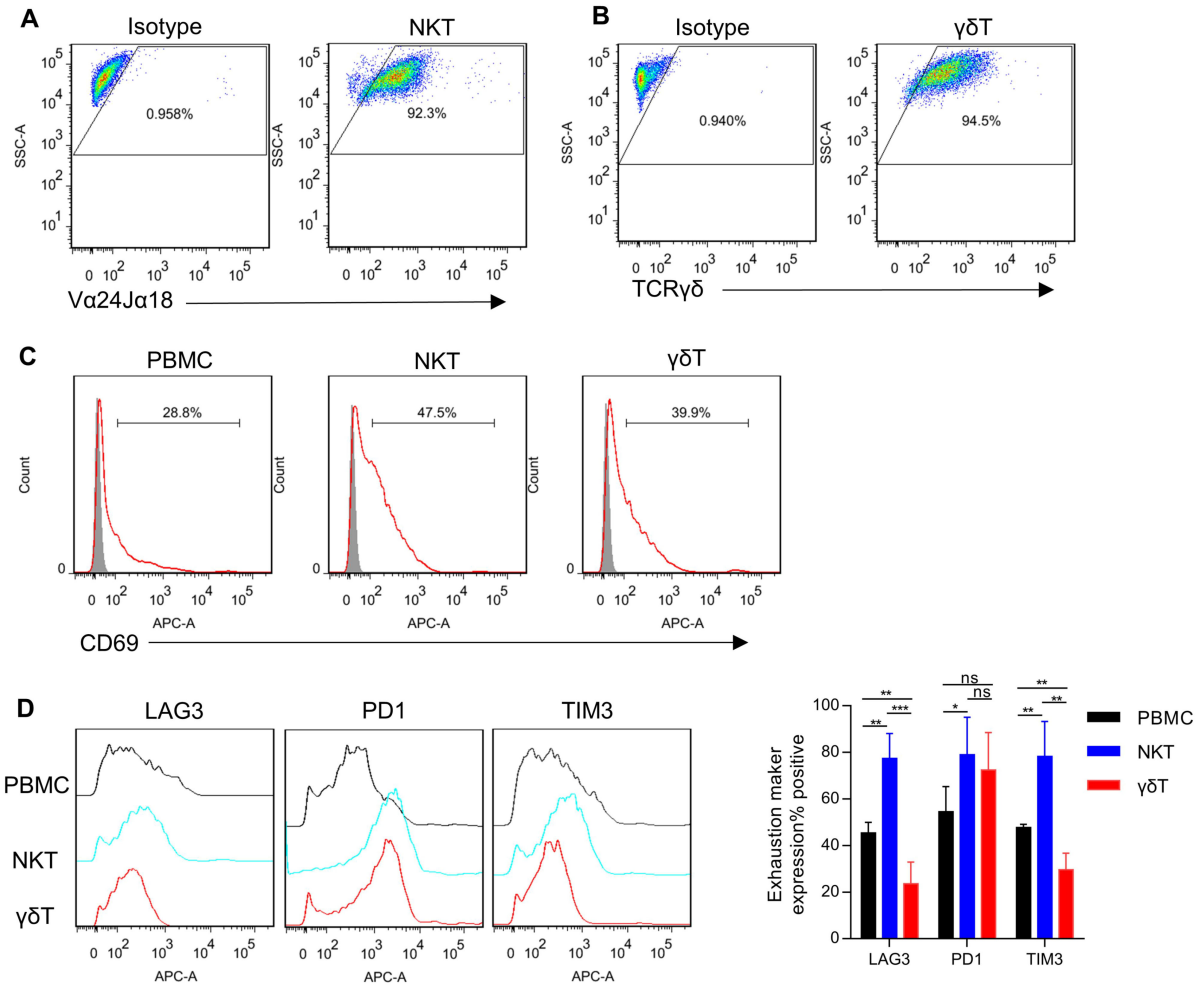
Generation of NKT and γδT cells. **(A)** NKT cells were isolated from human PBMCs, stimulated with α-GalCer, and expanded in culture with IL-2. The expanded cells were analyzed by FACS for the frequency of NKT cells using Vα24-Jα18 mAb (day 14 after stimulated). **(B)** γδT cells were isolated from human PBMCs, stimulated with Zoledronate, and expanded in culture with IL-2. The expanded cells were analyzed by FACS for the frequency of γδT cells using TCR gamma/delta antibody (day 14 after stimulated). **(C)** T cells (PBMCs, NKT, and γδT) and KLM-1 cells were incubated at a 10:1 ratio in the presence of MSLN/CD3 bispecific antibody (bsAb) for 24 hours. Following incubation, the expression of the activation marker CD69 on T cells was assessed using flow cytometry. **(D)** Surface expression of immune checkpoints after co-culture of PBMC、NKT or γδT cells with KLM-1 at an Effect/Target ratio of 1:1 in the presence of MSLN/CD3 bsAb for 72h. Data represent mean ± SEM(n=4). *p < 0.05, **p < 0.01, ***p < 0.001, ns, not significant, one-way ANOVA.

NKT cells were selectively expanded and activated from whole PBMCs using α-GalCer. On day 0, PBMCs were activated with α-GalCer, resulting in NKT cell-specific proliferation. By day 14, the purity of NKT cells exceeded 90% (**Figure 1A**). The focus of this study is the circulating γδT cells expressing Vγ9/Vδ2 TCR heterodimers, as they exhibit potent anti-tumor functions (34). Vγ9/Vδ2 T cells were selectively expanded and activated from whole PBMCs using Zoledronate. On day 0, PBMCs were also activated with Zoledronate, inducing γδT cell-specific proliferation. By day 14, the purity of γδT cells exceeded 90% (**Figure 1B**). In addition, after being stimulated by MSLN-positive KLM-1 cells and MSLN/CD3 bsAb, NKT cells and γδT cells showed higher percentage of CD69 expression than PBMC cells (**Figure 1C**). And NKT cells had significantly higher expression levels of PD-1, TIM3 and LAG3 than γδT cells and PBMC cells. γδT cells had significantly lower expression levels of TIM3 and LAG3 than NKT cells and PBMC cells (**Figure 1D**).

### *In vitro* cytotoxicity of the MSLN/CD3 bsAb with different subsets of T cells

**Figure 2:**
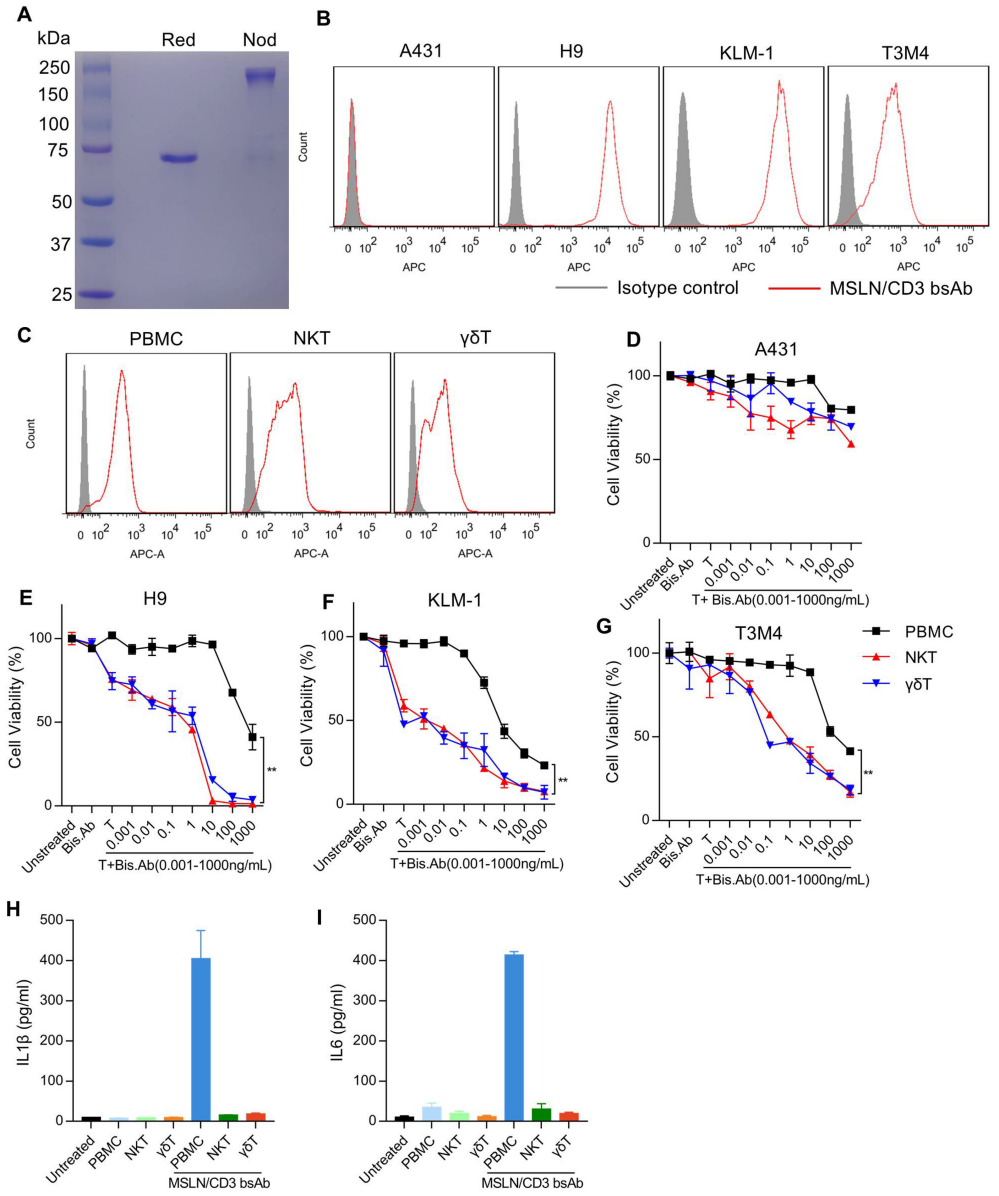
Binding ability and cytotoxicity analysis of the MSLN/CD3 bsAb with different subsets of T cells. **(A)** SDS-PAGE analysis of the purified MSLN/CD3 bsAb (R47-hFc-OKT3 format. Red, reduced condition, showing the reduced monomer of the MSLN/CD3 bsAb. Nod, non-reduced condition, showing the dimerized MSLN/CD3 bsAb). **(B)** The expression of MSLN in cancer cell lines was detected by flow cytometry. A431 cell was used as a negative control to demonstrate the specificity of the antibody, and H9 cell was used as a positive control. Two pancreatic cancer cell lines, KLM-1 and T3M4, were stained with 1 μg/mL of MSLN/CD3 bispecific antibody (bsAb), and the binding was detected with APC-conjugated goat anti-human polyclonal antibodies. **(C)** Flow cytometry was used to analyze the binding of different T cell subsets (PBMCs, NKT, or γδT) to the MSLN/CD3 bsAb. **(D-G)** An *in vitro* cell killing assay was performed using MSLN/CD3 bispecific antibody (bsAb) with different subsets of T cells against Luc-A431, Luc-H9, Luc-KLM-1 or Luc-T3M4 cells. Ten thousand cancer cells were incubated with one hundred thousand PBMCs, NKT, or γδT cells for 72 hours in the presence of varying concentrations of MSLN/CD3 bsAb. MSLN-negative A431 cells served as a control for antigen-independent, non-specific killing. Specific lysis was determined by normalizing the percentage of cell death to untreated cells. Data are presented as mean ± SEM. Statistical analysis was performed using an unpaired t-test, with p < 0.01 indicating significance. **(H, I)** The release of IL-1β and IL-6 from PBMCs, NKT cells, and γδT cells cultured with KLM-1 cells in the presence of 0.5 µg/ml MSLN/CD3 bispecific antibody was measured by ELISA (n=3). Untreated KLM-1 cells served as the control group.

The bispecific antibodies (MSLN/CD3 bsAb) were expressed in 293F cells and purified via protein A column (**Figure 2A**). The cell binding of the MSLN/CD3 bsAb was tested on MSLN-negative cell line A431, MSLN-overexpressing A431 cells (named H9), and MSLN-positive pancreatic cancer cell lines KLM-1 and T3M4, as well as different subsets of T cells (PBMC、NKT、γδT). As shown in **Figure 2B**, the MSLN/CD3 bsAb had specific binding to the MSLN-positive pancreatic cancer cell lines KLM-1, T3M4 and the MSLN-highly expressing H9 cells, but not to the MSLN-negative A431 cells (**Figure 2B**). The binding of MSLN/CD3 bsAb to T cells was confirmed on PBMC, NKT and γδT cells (**Figure 2C**). We evaluated the tumor killing and cytokine release mediated by the MSLN/CD3 bsAb in a co-culture assay using four MSLN+ tumor cell lines (KLM-1, T3M4 and H9) incubated with different subsets of T cells in the presence of the MSLN/CD3 bsAb. As shown in **Figures 2D–G**, MSLN/CD3 bsAb efficiently eradicated MSLN-positive KLM-1, T3M4, and H9 cells in a dose-dependent manner when co-cultured with PBMCs, NKT, and γδT cells *in vitro*, while exerting minimal impact on A431 cells. Additionally, both NKT and γδT cells demonstrated comparable anti-tumor activity, significantly superior to that of PBMCs (**Figures 2D–G**). PBMC in the presence of the MSLN/CD3 bsAb secreted high levels of IL-1β and IL-6, while NKT and γδT cells secreted low levels of IL-1β and IL-6 (**Figures 2H, I**). This also indicates that NKT and γδT cells have potential safety advantages in avoiding cytokine storm syndrome in cancer patients.

### *In vivo* cytotoxicity of the MSLN/CD3 bsAb with different subsets of T cells

**Figure 3:**
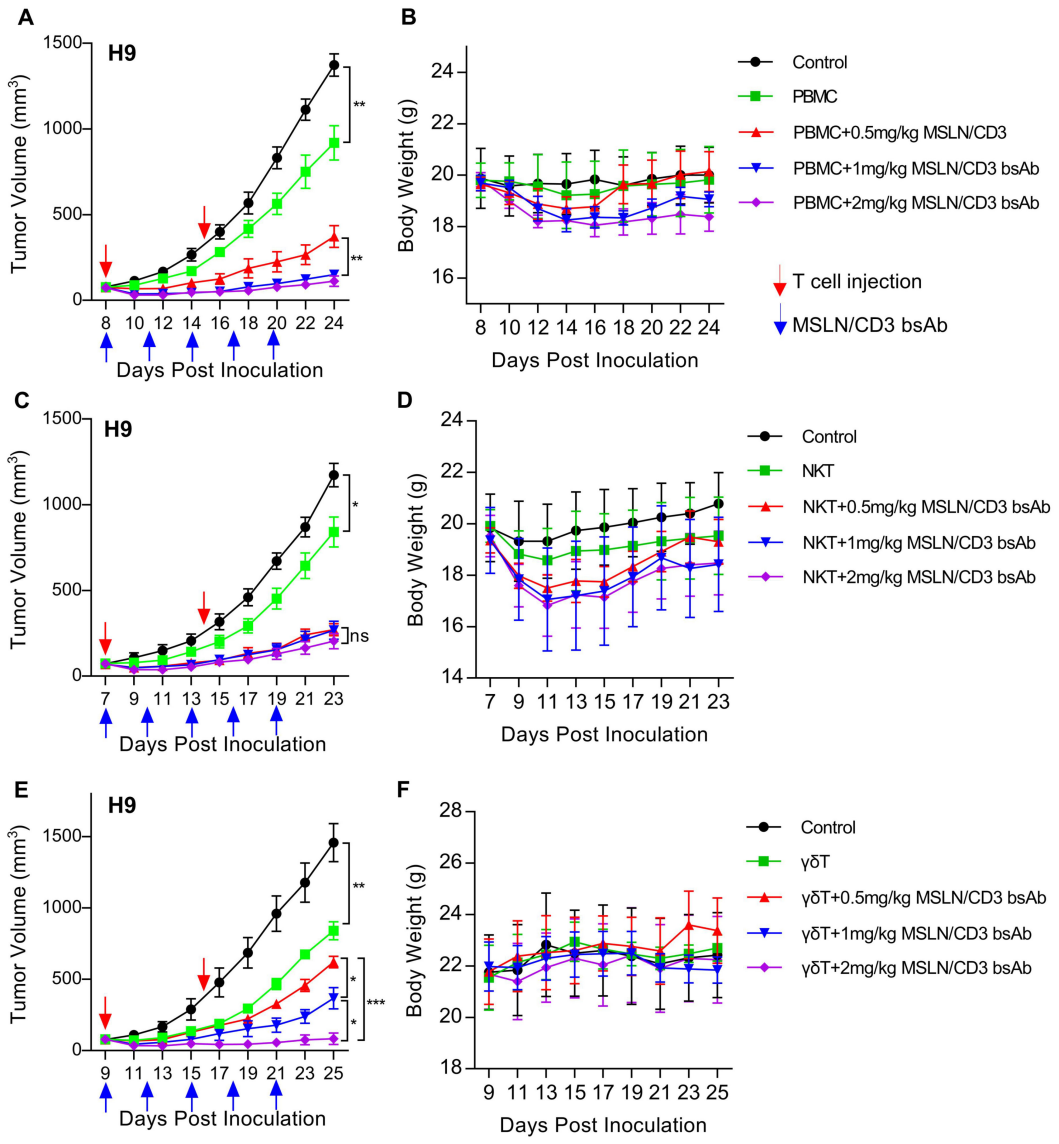
MSLN/CD3 bsAb induces different subsets of T cells antitumor activity *in vivo*. **(A, B)** Tumor growth inhibition and body weight was evaluated in H9 tumor bearing NSG mice comparing treatment with different concentrations of MSLN/CD3 bsAb using PBMC as effectors cells. **(C, D)** Tumor growth inhibition and body weight was evaluated in H9 tumor bearing NSG mice comparing treatment with different concentrations of MSLN/CD3 bsAb using NKT as effectors cells. **(E, F)** Tumor growth inhibition and body weight was evaluated in H9 tumor bearing NSG mice comparing treatment with different concentrations of MSLN/CD3 bsAb using γδT as effectors cells. Blue arrows indicated the injection time point of the MSLN/CD3 bsAb. Red arrow indicates the injection time point of T cell. Data represent as the mean ± SEM(n=6). Statistical analysis for **(A, C, E)** was performed using a two-way ANOVA. *p < 0.05, **p < 0.01, ***p < 0.001, ns, not significant.

To evaluate the *in vivo* tumor suppression activity of the MSLN/CD3 bsAb in the presence of PBMC, NKT and γδT, a xenograft mouse model was established by subcutaneous transplantation of H9 cells in NSG mice. When the tumor reached the size of 100-200 mm^3^, treatment was initiated by tail vein injection of 5 million PBMC, NKT or γδT once a week and tail vein injection of 0.5, 1 or 2 mg/kg of the MSLN/CD3 bsAb every two days. As shown in **Figures 3A, B**, the MSLN/CD3 bsAb significantly inhibited the tumor growth of H9 cells in the presence of PBMC, and this effect was dose-dependent (**Figure 3A**). The body weight of the treated mice also significantly decreased, and this was correlated with the dose of the bispecific antibody (**Figure 3B**). As shown in **Figures 3C, D**, the MSLN/CD3 bsAb significantly inhibited the tumor growth of H9 cells in the presence of NKT, and this effect was independent of the dose (**Figure 3C**). The body weight of the treated mice in this study also significantly decreased, independent of the dose of the bispecific antibody (**Figure 3D**). As shown in **Figures 3E, F**, the MSLN/CD3 bsAb significantly inhibited the tumor growth of H9 cells in the presence of γδT, and this effect was dose-dependent (**Figure 3E**). The body weight of the treated mice did not change (**Figure 3F**).

### γδT cells have stronger and safer anti-tumor activity

**Figure 4:**
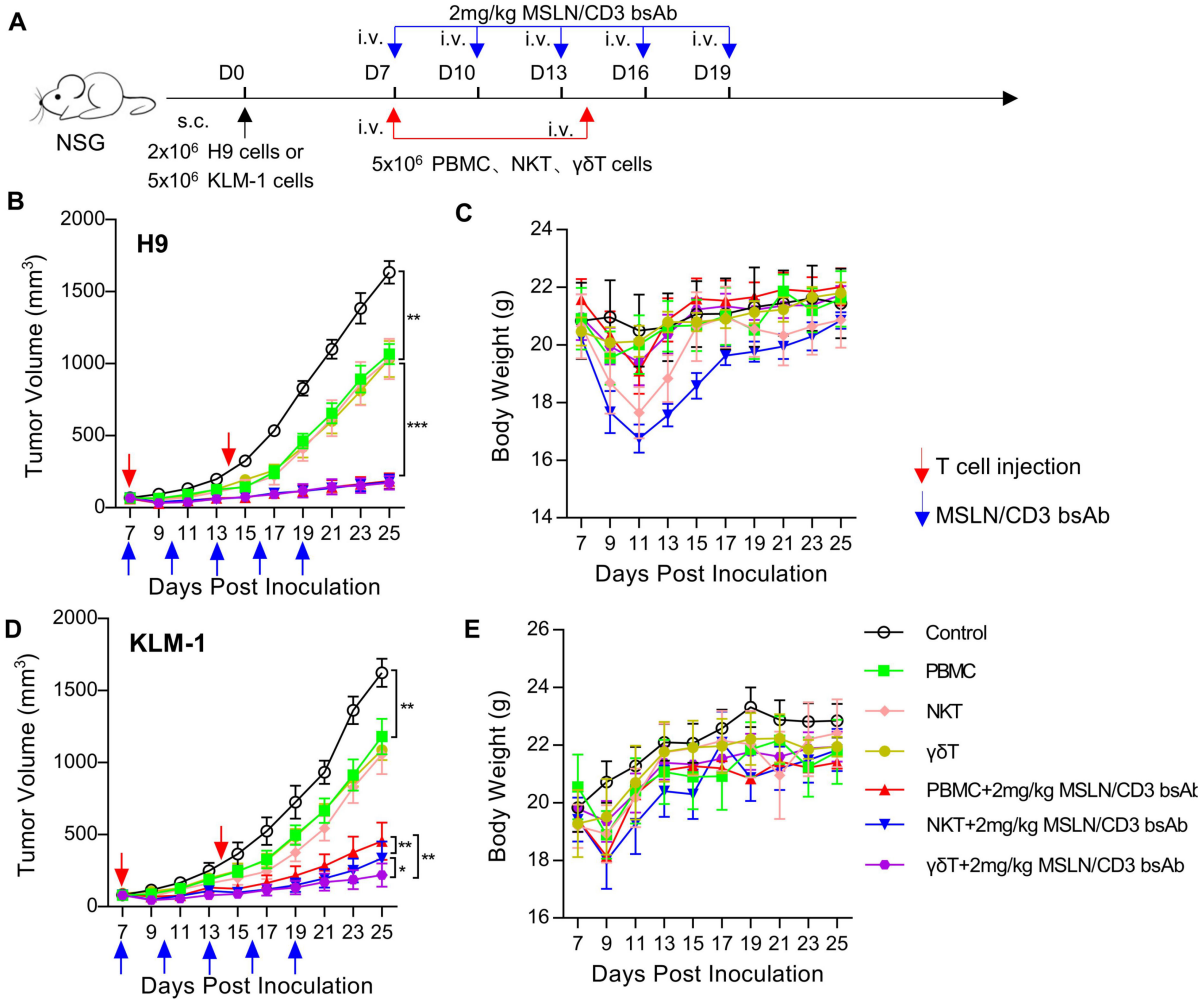
Comparison of *in vivo* activity of different subsets of T cells. **(A)** Schematic representation of the study design and timeline of the treatment. NCG mice were implanted with 2 × 10^6^ H9 cells or 5 × 10^6^ KLM-1 cells in the right flank on day 0. On day 7, the mice received i.v. infusion of 5 × 10^6^ T cells (PBMC、NKT、γδT) once a week and i.v. administration of 2 mg/kg MSLN/CD3 bsAb every 3 days for five times. Blue arrows indicated the injection time point of the MSLN/CD3 bsAb. Red arrow indicates the injection time point of T cell. **(B)** Tumor growth curve of H9. **(C)** Body weight of the H9 mouse model. **(D)** Tumor growth curve of KLM-1. **(E)** Body weight of the KLM-1 mouse model. Data represent as the mean ± SEM(n=6). Statistical analysis for **(B, C)** was performed using a two-way ANOVA. *p < 0.05, **p < 0.01, ***p < 0.001.

To evaluate the *in vivo* tumor suppression activity of PBMC, NKT and γδT, a xenograft mouse model was established by subcutaneous transplantation of H9 or KLM-1 cells in NSG mice (**Figure 4A**). In the H9 tumor model, PBMC, NKT and γδT significantly inhibited the tumor growth in the presence of the MSLN/CD3 bsAb (**Figure 4B**), However, the body weight of the NKT-treated mice decreased the most, followed by the PBMC-treated mice, while the γδT-treated mice maintained stable body weight (**Figure 4C**). In the KLM-1 tumor model, NKT and γδT significantly inhibited the tumor growth in the presence of the MSLN/CD3 bsAb better than PBMC, with γδT showing the best anti-tumor effect (**Figure 4D**). The body weight of the treated mice was similar to that in the H9 tumor model (**Figure 4E**). The disparity in activity observed between the H9 and KLM-1 models could be attributed to the origin of the cells; H9 cells are derived from A431 cells overexpressing MSLN and thus exhibit higher resistance, whereas KLM-1 cells are more susceptible to immune responses. Furthermore, the findings also suggest that γδT cells possess stronger and safer anti-tumor activity.

### The effects of different subsets of T cells on mouse organs

**Figure 5:**
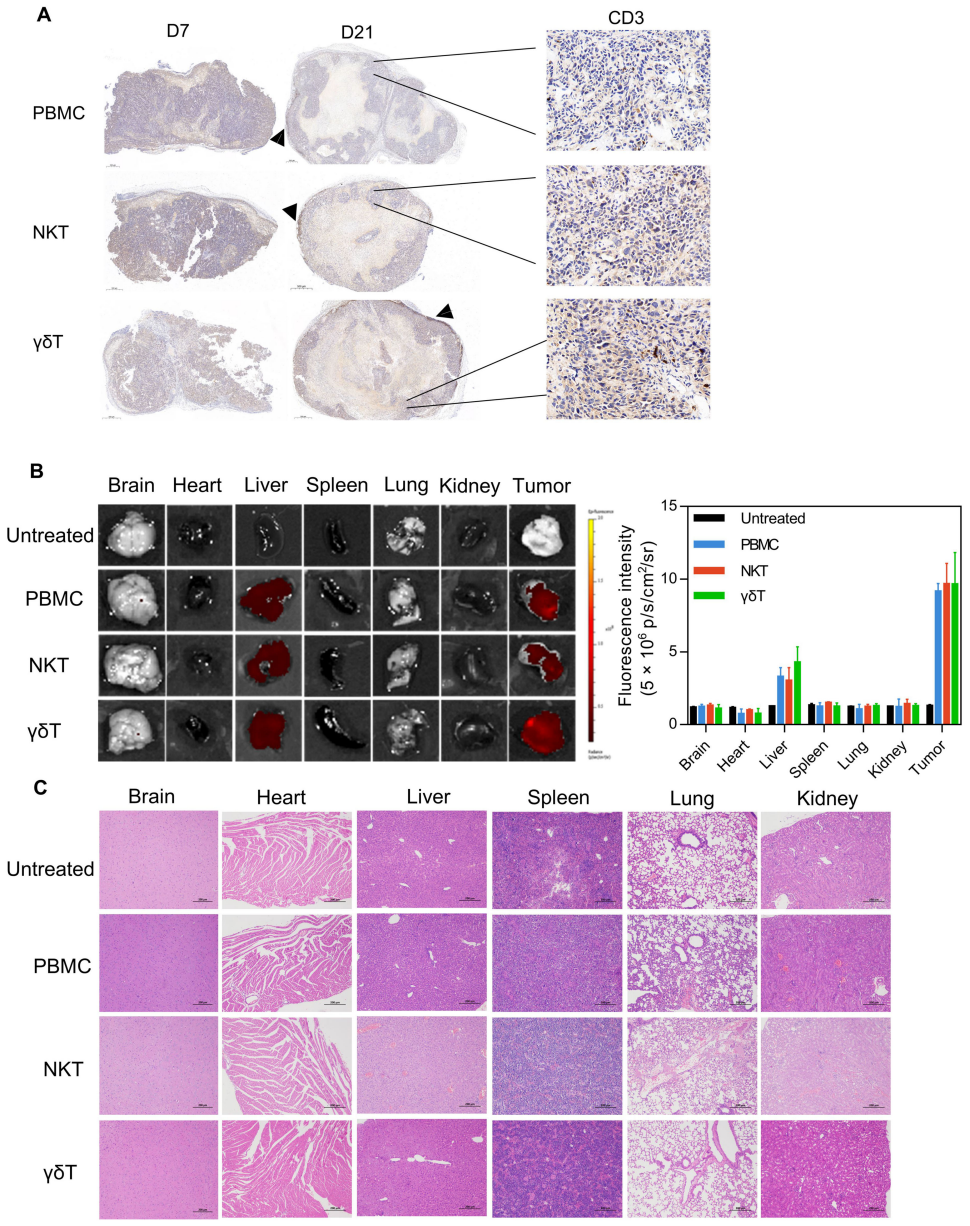
The infiltration of NKT and γδT cells T cells, and toxicity side effects. **(A)** KLM-1 tumors were engrafted subcutaneously, treated with different subsets of T cells and 2 mg/kg MSLN/CD3 bsAb, and analyzed by IHC for T cell infiltration (anti-CD3 stain). Tumors were collected 7 days (left) and 21 days (center) after T cell injection. Zoomed out scale bars are 500 microns, zoomed in are 50 microns. **(B)** Fluorescence images and fluorescence intensity of main organs from ex vivo at 24h after i.v. injection of PBMC、NKT or γδT cells. **(C)** Organs of mice treated with different subsets of T cells and 2 mg/kg MSLN/CD3 bsAb. Hematoxylin and eosin (H&E) staining of vital organs. This study performed H&E staining on organs such as heart, liver, spleen, lung and kidney. Specimens were obtained from KLM-1 mice that we sacrificed at the end of the study. The scale bar represents 200 µm. The data shown are representative of experiments with similar results. The images were obtained at 200x magnification.

To better describe the control of different subsets of T cells on the pancreatic cancer model, we performed a more in-depth analysis of the tumors during the treatment. We collected the early and regressed specimens of KLM-1 pancreatic tumors treated with different subsets of T cells at 7 and 21 days after treatment, and measured the infiltration of CD3+ T cells by immunohistochemistry. At 7 days after treatment, T cells were enriched in the tumors treated with different subsets of T cells. At 21 days after treatment, the tumors treated with PBMC cells showed a typical immune rejection phenotype, with very limited T cell infiltration in the tumor core and most T cells accumulated at the tumor periphery. In contrast, the tumors treated with NKT and γδT cells had much more T cell infiltration throughout the tumor core (**Figure 5A**). To investigate the homing ability of PBMC, NKT and γδT cells to the tumor under the effect of MSLN/CD3 bsAb, PBMC, NKT and γδT cells labeled with Dil dye were transferred into mice bearing established KLM-1 tumors (about 1000 mm^3^), and 2 mg/kg MSLN/CD3 bsAb was injected at the same time. After 24 hours, the harvested organs confirmed that PBMC, NKT, and γδT cells mainly homed to the tumor, although a small fraction of these cells was also found in the liver of some mice (**Figure 5B**). Additionally, this study investigated whether PBMCs, NKT cells, and γδT cells would cause toxicity to healthy tissues in mice. Various organs such as the brain, heart, liver, spleen, lungs, and kidneys were extracted from treated mice and subjected to HE staining. Histological analysis revealed that mice treated with PBMCs and NKT cells exhibited lung injury, as evidenced by H&E staining showing severe damage to alveolar capillary structures and inflammatory cell infiltration. Furthermore, mice treated with NKT cells showed liver and kidney damage, while mice treated with PBMCs exhibited kidney damage. However, mice treated with γδT cells did not exhibit any significant damage (**Figure 5C**). These results indicate that γδT cells have stronger anti-tumor effects than PBMCs, and can effectively accumulate and survive in the tumor without causing harm to vital organs.

### Comparison of *in vivo* activity of preactivated NKT and γδT cells

**Figure 6:**
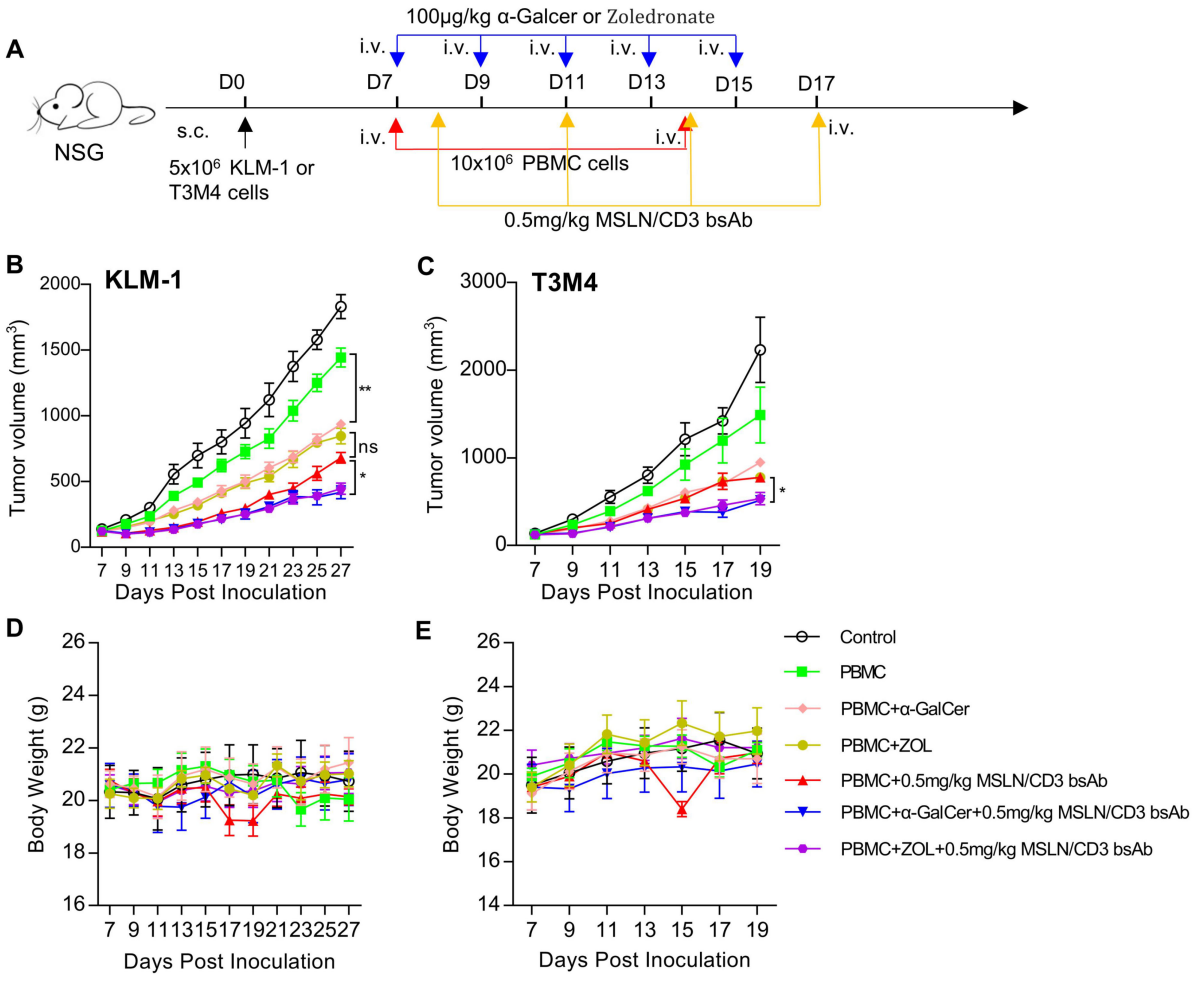
Comparison of *in vivo* activity of preactivated NKT and γδT cells. **(A)** Schematic representation of the study design and timeline of the treatment. NCG mice were implanted with 5×10^6^ KLM-1 or T3M4 cells in the right flank on day 0. On day 7, the mice received i.v. infusion of 10x10^6^ PBMC cells once a week and i.v. administration of 100 μg/kg α-Galcer or Zoledronate every other day for five times. On day 8, the mice were i.v. treated with 0.5mg/kg MSLN/CD3 bsAb every 3 days for four times. Blue arrows indicated the injection time point of the MSLN/CD3 bsAb. Red arrow indicates the injection time point of PBMC cells. Yellow arrow indicates the injection time point of α-Galcer or Zoledronate. **(B)** Tumor growth curve of KLM-1. **(C)** Tumor growth curve of T3M4. **(D)** Body weight of the KLM-1 mouse model. **(E)** Body weight of the T3M4 mouse model. Data represent as the mean ± SEM(n=6). Statistical analysis for **(B, C)** was performed using a two-way ANOVA. ns, not significant. *p < 0.05, **p < 0.01.

This study used intraperitoneal injection of α-Galcer or Zoledronate to activate or expand NKT or γδT cells *in vivo*, to further verify the anti-tumor effects of NKT and γδT cells. In brief, KLM-1 or T3M4 cells were subcutaneously injected into NSG mice to form palpable solid tumors. Treatment was initiated by intravenous injection of human PBMCs, followed by intravenous injection of α-Galcer or Zoledronate every other day. MSLN/CD3 bsAb was intravenously injected every 3 days (**Figure 6A**). As shown in **Figures 6B, C**, compared with the control and PBMC alone, tumor regression was observed in mice after injection of α-Galcer or Zoledronate. In contrast to PBMC+ MSLN/CD3 bsAb, injection of α-Galcer or Zoledronate combined with MSLN/CD3 bsAb treatment showed significant and lasting tumor growth inhibition, and the anti-tumor effects were similar. In addition, this study also measured the body weight of the mice during all treatments. Except for the PBMC+MSLN/CD3 bsAb treatment group, which showed obvious fluctuations in body weight during treatment, there was no significant difference in body weight between the other groups (**Figures 6D, E**), indicating that α-Galcer or Zoledronate induced NKT or γδT cells did not cause systemic toxicity to the mice.

## Discussion

T cell-targeted therapy [chimeric antigen receptor (CAR) T and CD3 bispecific antibody] is an emerging medical advancement, but there are many unresolved questions about its optimal clinical application, such as how to manage CRS. Evidences suggest that the main cause of CRS induced by CD3-bispecific antibodies (CD3-BsAbs) is the release of TNF-α by activated T cells, which leads to monocyte activation and the production of systemic toxic cytokines (35). By inhibiting TNF-α and its downstream IL-1β or IL-6, CRS symptoms can be alleviated (35–39). Additionally, some studies have shown that lowering the affinity of CD3 or switching to subcutaneous administration can decrease the cytokine levels caused by treatment and also reduce the incidence of CRS (40, 41).

Previous studies have demonstrated the potential of CAR-NKT cells in cancer therapy, showing that NKT cells can effectively target and kill tumor cells with minimal side effects (42). Similarly, γδT cells have been shown to possess strong anti-tumor activity and the ability to infiltrate solid tumors (43). Building on these findings, this study developed a strategy utilizing MSLN/CD3 bsAb to activate and recruit specific subsets of NKT or Vγ9Vδ2 T cells, thereby effectively identifying and eliminating MSLN-positive tumor cells. *In vitro* experiments demonstrated that, compared to using PBMCs, NKT and γδT cells significantly enhanced the cytotoxicity of MSLN/CD3 bsAb against MSLN-positive tumor cells while releasing lower levels of pro-inflammatory cytokines IL-1β or IL-6, helping to reduce the risk of cytokine release syndrome.

In solid tumor therapy, CD3-BsAbs are a promising immunotherapy that can redirect T cells to tumor cells, but they face the major challenge of the lack of effective T cells and T cell exhaustion in the tumor microenvironment. To address this issue, this study explored the potential of NKT and γδT cells as synergistic therapeutic cells for CD3-BsAbs (44–46). This research established a mouse xenograft model by injecting human pancreatic cancer cells into immunodeficient mice, and then administered MSLN/CD3 BsAb and human T cells from different sources (PBMC, NKT, or γδT cells). This research found that, compared with PBMC, NKT and γδT cells were able to infiltrate and survive more efficiently in the tumor tissue, and significantly enhanced the antitumor effect of MSLN/CD3 BsAb on pancreatic cancer. Moreover, this research observed by *in vivo* imaging that the infused NKT and γδT cells selectively homed to the tumor site, providing an additional advantage for their synergistic action in the presence of MSLN/CD3 BsAb.

This study found that NKT-mediated CD3-BsAb could more effectively activate and recruit NKT cells, thereby enhancing their cytotoxicity against tumor cells. However, this therapeutic strategy also had some adverse effects, including significant weight loss, organ dysfunction, and tissue damage in the lungs, liver, and kidneys of mice. Some studies have shown that excessive activation of NKT cells (47–50). Therefore, to reduce the toxicity of NKT-mediated CD3-BsAb, appropriate modulation strategies are needed, such as optimizing the dose, frequency, and route of administration, or combining with other anti-inflammatory or immunomodulatory drugs. Surprisingly, γδT-mediated CD3-BsAb treatment did not affect the weight and organs of mice, but rather had a stronger anti-tumor effect. There are also reports that γδT cells do not cause damage to normal organ cells (51).

Early prospective trials have found that Zoledronate combined with IL-2 can effectively activate Vγ9Vδ2 T cells *in vivo* and inhibit various malignant tumors (52). In addition, some studies have also explored the role of Zoledronate in adoptive cell transfer, and found that Zoledronate administration systemically or locally can enhance the anti-tumor activity of Vγ9Vδ2 T cells without additional IL-2 (53–55). Morita et al. reported that injection of α-GalCer after subcutaneous inoculation of B16 cells on days 1, 5, and 9 reduced tumor volume by about 50% (56). Gehrmann et al. showed that α-GalCer-loaded exosomes derived from dendritic cells could effectively suppress tumor growth and prolong mouse survival (57). This research study results also found that systemic administration of α-GalCer or Zoledronate could enhance the anti-tumor effect of MSLN/CD3 bsAb, and no obvious toxicity was observed. However, this study also has some limitations. This research did not utilize *in situ* tumor models and wild-type mice to investigate the anti-tumor efficacy and toxicity of α-GalCer or Zoledronate *in vivo* for activating NKT or γδT cells. Instead, it employed an immunodeficient mouse model, which does not fully capture the complexity and functionality of the immune system. Moreover, this research did not compare the use of CD3-BsAb to activate and recruit NKT or γδT cells with the use of NKT or γδT cell-specific antibodies. Of course, there are also studies that show that NKT or γδT cell-specific antibodies have similar anti-tumor effects (58–60).

Previous studies have demonstrated the potential of CAR-NKT cells in cancer therapy, showing that NKT cells can effectively target and kill tumor cells with minimal side effects. Similarly, γδT cells have been shown to possess strong anti-tumor activity and the ability to infiltrate solid tumors. This study builds on these findings by demonstrating that NKT and γδT cells can enhance the efficacy of CD3-BsAbs while reducing the risk of CRS, a significant limitation of current T cell-targeted therapies.

In summary, our results demonstrate that NKT and γδT cells are a promising synergistic therapeutic cell type that can overcome the limitations of CD3-bispecific antibodies in solid tumor treatment, providing new insights for the clinical translation in this field.

## Data Availability

The original contributions presented in the study are included in the article/supplementary material. Further inquiries can be directed to the corresponding author/s.
